# Molecular mechanisms and clinical applications of exosomes in prostate cancer

**DOI:** 10.1186/s40364-022-00398-w

**Published:** 2022-07-29

**Authors:** Xiaolin Cui, Qiang Fu, Xueying Wang, Pengcheng Xia, Xianglun Cui, Xiaohui Bai, Zhiming Lu

**Affiliations:** 1 Department of Clinical Laboratory Medicine, Shandong Provincial Hospital Affiliated to Shandong First Medical University, Jinan, 250021 Shandong China; 2 Department of Clinical Laboratory, Shandong Provincial Hospital, Cheeloo College of Medicine, Shandong University, Jinan, 250021 Shandong China; 3 Department of Urology, Shandong Provincial Hospital Affiliated to Shandong First Medical University, Jinan, Shandong China

**Keywords:** Prostate cancer, Exosome, Epithelial-mesenchymal transition, Angiogenesis, Tumor microenvironment, Drug resistance, Biomarker, Targeted therapy

## Abstract

Prostate cancer (PC) is a common tumor in men, and the incidence rate is high worldwide. Exosomes are nanosized vesicles released by all types of cells into multiple biological fluid types. These vesicles contribute to intercellular communication by delivering both nucleic acids and proteins to recipient cells. In recent years, many studies have explored the mechanisms by which exosomes mediate the epithelial-mesenchymal transition, angiogenesis, tumor microenvironment establishment, and drug resistance acquisition in PC, and the mechanisms that have been identified and the molecules involved have provided new perspectives for the possible discovery of novel diagnostic markers in PC. Furthermore, the excellent biophysical properties of exosomes, such as their high stability, high biocompatibility and ability to cross biological barriers, have made exosomes promising candidates for use in novel targeted drug delivery system development. In this review, we summarize the roles of exosomes in the growth and signal transmission in PC and show the promising future of exosome contributions to PC diagnostics and treatment.

## Background

As the leading cause of cancer deaths in men in 46 countries, prostate cancer (PC) is the most common tumor in men in 105 of 185 countries [1]. In recent years, the incidence of PC has increased worldwide [2].

According to the current clinical guidance [3], successful screening as well as early diagnosis and accurate risk assessment for PC are highly dependent on the detection of prostate-specific antigen (PSA), which remains the most commonly used PC biomarker [4]. Although the beneficial effects of PSA testing have been confirmed in a long-term multicenter follow-up study [5–8], the poor specificity of PSA tests has resulted in many misdiagnoses, and the negative effect of this insurmountable problem on treatment monitoring has continuously worn down the patience of physicians and patients [9–11]. Fortunately, the development of the PSA-based Prostate Health Index (PHI) [12] and the 4KScore [13], as well as the implementation of noninvasive PCA3 and TMPRSS2:ERG gene fusion screening, has had positive impacts on the accuracy of PC diagnostics. However, the true value of these discoveries has yet to be determined with large-scale prospective comparative studies [3]. Although significant advancements have been made in early PC management and in the use of radiation and chemotherapy in recent years, these clinical strategies are effective only for the local treatment of PC and show little impact on metastatic PC [3, 14].

As extracellular vesicles, exosomes range from 30 to 200 nm in diameter, with an average diameter of 100 nm [15, 16]. They are organelles with a single membrane and contain abundant proteins, lipids, nucleic acids, and glycoconjugates [17]. Exosomes are secreted by all cell types [18] and are involved in physiological and pathological processes by acting as carriers for intercellular substance exchange and/or signaling [19–23]. In recent years, the role played by exosome intercellular communication in PC development has gradually been demystified. Furthermore, excellent biophysical properties render exosomes promising for use as novel PC biomarkers and for developing new treatments [24, 25].

In this review, we summarize the potential mechanisms by which exosomes contribute to PC infiltration and metastasis, mediate drug resistance, and structure the tumor microenvironment. Furthermore, the biogenesis of exosomes and the regulatory factors of this biogenesis; the isolation and characterization of exosomes; the roles played by exosomes as biomarkers in PC screening, diagnosis, disease stratification, and treatment evaluation; and use of exosomes as therapeutic vectors in cutting-edge advancements for targeting PC are illustrated in this review.

## Exosomes

### Biogenesis of exosomes

**Fig. 1:**
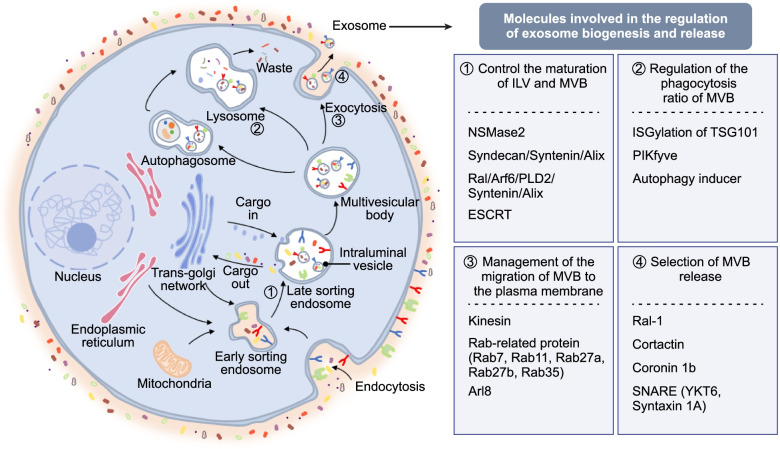
Biogenesis of exosomes and their regulatory factors

The best characterized pattern of exosome biogenesis is the endosomal pathway, which involves two separate instances of plasma membrane invagination and the generation of intracellular multivesicular bodies (MVBs) (Fig. 1). Although it is a continuous process, the endosomal pathway is generally divided into four main steps to enable better understanding: In step 1, various extracellular substances and fluids enter a cell via plasma membrane invagination or endocytosis, leading to the expression of cell surface proteins (e.g., CD9, CD63, CD81, and flotillin) in the inner layer of the exosomal phospholipid membrane, resulting in plasma membrane budding, and these budded vesicles either form early sorting endosomes (ESEs) or fuse with previously formed ESEs in a process regulated by endoplasmic reticulum (ER), trans-Golgi network (TGN), and/or mitochondrial components. In step 2, facilitated by the TGN, ESEs mature into late sorting endosomes (LSEs). In step 3, the plasma membrane invaginates at multiple LSE sites, leading to the formation of MVBs, which contain multiple small vesicles, ~ 40 nm in diameter, called intraluminal vesicles (ILVs). In step 4, MVBs either bind to autophagosomes or lysosomes or fuse with the plasma membrane, and their cargo is either degraded or released into the extracellular space. Specifically, ILV binding with either autophagosomes or lysosomes leads to the eventual degradation ILVs, which are then prone to extracellular disposal, and MVB fusion with the plasma membrane leads to extracellular secretion of MVB cargo and ILV development into true exosomes [15, 26, 27]. The endosomal pathway has been verified through many studies [28–30]. However, it has become increasingly clear that exosomes can directly bud from the plasma membrane. The original mechanism, in which ILVs become exosomes, was first challenged by the discovery of the plasma membrane as the primary site of HIV-1 outgrowth [31]. Subsequently, microvesicles (MVEs) germinated directly from the plasma membrane are the same size as exosomes but lack LSE markers, suggesting that these vesicles differ from exosomes derived from MVBs [32]. The application of atomic force microscopy in recent years has provided a direct view of this germination pathway [33].

### Regulatory factors of exosome biogenesis and release

The biogenesis of exosomes is tightly controlled by multiple proteins and the networks in which these proteins are involved. Exploration into the molecular mechanisms regulating exosome biogenesis has provided a theoretical basis for the search for potential PC markers and therapeutic targets. Here, we review the regulatory factors involved in the four main steps of the endosomal pathway and exosome biogenesis (Fig. 1).

Step 1. Factors affecting ILV formation and MVB maturation. The endosomal sorting complex required for transport (ESCRT) is the main mechanism regulating ILV production and is critical for sorting ubiquitinated proteins into ILVs, indicating that exosome production in the endosomal pathway is dependent on functional ESCRT-related proteins [34, 35]. As an auxiliary component of the ESCRT machinery, Alix is a pivotal molecule for the Syntecan/Syntenin/Alix axis [36], the Ral/Arf6/PLD2/Syntenin/Alix axis [16, 37] to produce cross-linking with ESCRT and plays an important role in influencing exosomes [38]. Furthermore, ceramide plays a key role in the biogenesis of MVBs. For example, neutral sphingomyelinase 2 (NSMase2) hydrolysis of sphingolipids produces ceramides that enable ILVs to form MVBs; notably, exosome production was found to be decreased when NSMase2 activity was inhibited [39].

Step 2. Factors enhancing the destruction of MVBs. Interferon-stimulated gene conjugation (ISGylation) to TSG101 induces degradation of MVBs by aggregating and increasing MVB binding to lysosomes, decreasing the number of exosomes secreted [40]. Notably, when the expression of PIKfyve in PC-3 cells was blocked, the secreted exosomes were profoundly enriched with autophagy-related proteins [41]. Furthermore, autophagy inducers, such as starvation or rapamycin, promoted the fusion of MVBs with autophagic vesicles, leading to a decrease in exosome release [42].

Step 3. Mature MVBs are induced to participate in autophagy, bind to lysosomes, or are transported along microtubules to the plasma membrane [27]. This transport is driven by kinesin [43] and regulated by several Rab proteins [28] and the small GTPase Arl8 [44]. The key roles of the Rab protein family in the biogenesis of exosomes have been extensively explored, and studies have shown, for example, that Rab11 promoted homotypic fusion of MVBs in a calcium-dependent manner [45], Rab27a and Rab27b play roles in MVB docking to the plasma membrane [28], and inhibition of Rab35 activity leads to increased accumulation of endosomal vesicles [46].

Step 4. Docking and fusion are the final steps in exosome biogenesis prior to their release. After migration to the plasma membrane, MVBs couple with SNARE localized on the intracellular membrane and fuse to the plasma membrane [47]. This process is regulated by Ras-related GTPase homolog (Ral-1) [37]. Both YKT6 [47] and Syntaxin 1A [48] are SNAKE proteins and play irreplaceable roles in MVB docking. Moreover, the binding of Rab27a to cortactin and coronin 1b plays a key role in maintaining the stability of the MVE docking site on cortical actin [49].

### Exosome isolation and characterization

Exosomes are generally isolated from a complex variety of biological fluids, including blood, lymph, urine, cerebrospinal fluid, bile, saliva, milk, and amniotic fluid [50]. Many novel exosome isolation techniques have been developed, and they are more efficient than the original ultracentrifugation method. However, the complete purification of exosomes is still difficult, and each technical approach has advantages and limitations. Therefore, the term isolation is used to describe the process of harvesting as many exosomes as possible [51]. Generally, it is recommended that multiple types of technical approaches be used in combination to isolate exosomes (Table 1). After exosome isolation, identification techniques are promptly used to confirm the specific collection of exosomes (Table 1). Exosomes are commonly described on the basis of their size, shape, density, and markers [15], and exosome identification techniques are based on the biophysical properties of these characteristics [64]. Under cryo-electron microscopy, the diameter of only one-half of all types of vesicles fall in the range of 30-200 nm, which is generally considered the exosome diameter size. Although the shape of exosomes is usually regular, endosomes with multiple membranes and/or complex shapes are frequently observed [16]. Furthermore, the density of exosomes is affected by their protein content [71], and the characterization of surface markers reflects only the specific origins of the exosomes and are therefore not broadly representative [16]. It is clear that the characterization methods based on either exosome morphology and size lead to biased results, suggesting that characterization methods should be combined for the identification of exosomes.

**Table 1:** Techniques for exosome isolation and identification and their advantages and disadvantages

Separation technology		Advantages	Disadvantages	Refs
**Exosome isolation techniques**				
Centrifugation techniques	Ultracentrifugation (UC)	First used Well-developed	Low purity	[52, 53]
	Density gradient centrifugation	Higher purity than UC	Low yield Time-consuming	[54, 55]
Size-based techniques	Ultrafiltration	High purity High particle yield	Exosome damage Time-consuming	[50, 53, 56]
	Size exclusion chromatography	Reproducible Cost-effective Nondestructive	High workload Possible contamination	[50, 57]
Capture-based techniques	Magnetic beads and immunoaffinity	High purity Specific isolation Time save	Not high-throughput High cost Low yield Only specifically labeled exosomes are isolated	[50, 58]
	Heparin affinity	Wide range of affinity High recovery	Possible contamination Unspecified mechanism	[53, 59]
Precipitation	Polyethylene glycol precipitation	Suitable for commercial kits Convenient operation	High contamination Unstable results	[53, 60, 61]
Microfluidic systems	Based on size, density, immunoaffinity, and additional novel sorting mechanisms	Efficient acquisition Continuous separation with small samples	High requirements for equipment	[62, 63]
**Exosome identification techniques**				
Size-based technology	Nanoparticle tracking analysis	Fast detection High lower-detection limit	Difficult to distinguish similarly sized impurities	[64, 65]
	Tunable resistive pulse sensing	Fast detection	Poor specificity	[65]
	Dynamic light scattering	Fast detection High lower-detection limit	Applicable only to transparent solutions	[66, 67]
Morphology-based technology	Transmission electron microscopy	High accuracy Internal structures visible	Complex operation Possible deformation caused by preprocessing Low through-put	[16, 68]
	Scanning electron microscopy	High accuracy Surface structures are visible		[69]
	Atomic force microscopy Cryo-electron microscopy	High accuracy	Costly equipment, Low-throughput	[16]
Marker protein-based technology	Western blotting	Technology maturity Low threshold	For single marker	[64]
	ELISA	High specificity High through-put	Complex operation Time-consuming	[64]
	Flow cytometry	High through-put	Heavy reliance on high-level operations High lower-detection limit	[70]

## Involvement of exosomes in prostate cancer progression

Exosome transport of cellular cargoes between cells is an intercellular communication mechanism in which the contents of a donor cell are transferred to a recipient cell to functionally regulate the recipient cell [72]. Increasing evidence has shown that the progression and metastasis of PC depends on intercellular communication based on exosomes [73, 74].

PC progresses through a combination of the following medical conditions: (1) The epithelial-mesenchymal transition (EMT) in PC leads to the loss of characteristic epithelial cell adhesion and thus PC cells acquire invasive capacity [73, 75]. (2) Angiogenesis creates suitable nutritional conditions for tumor invasion [76]. (3) The generation of a microenvironment suitable for tumor growth promotes the growth and spread of tumor cells [76]. As important extracellular matrix (ECM) components, both cancer-associated fibroblasts and tumor-associated macrophages enhance tumor cell invasion by secreting inflammatory factors and promoting the tumor stem cell proliferation [77]. (4) The acquisition of drug resistance leads to the rapid failure of antitumor drugs and enhances overall tumor drug resistance [78]. In recent years, studies have revealed the involvement of exosomes in the aforementioned mechanisms underlying PC progression (Table 2).

**Table 2:** Involvement of exosomes in the progression of prostate cancer (PC)

Type	Cargo	Donor cells	Recipient cell	Targets	Application	Year	Ref.
**Exosomes contribute to epithelial-mesenchymal transition (EMT) in PC.**							
protein	integrin alpha 2 subunit	PC-3	LNCaP, C4-2B, RC77T/E	FAK, ERK1/2	Induces the EMT	2020	[79]
protein	Prostate-specific G-protein-coupled receptor	PC-3	hFOB1.19	E-cadherin, Vimentin, Snail, SOX2, OCT4a	Promotes migration, invasion, stemness, and EMT	2020	[80]
protein	Caveolin-1	LNCaP, Du145	LNCaP, Du145	NFκB	Promotes tumor stem cell phenotype acquisition and the EMT	2019	[81]
miRNA)	miR-100-5p,miR-21-5p	Bulk cells, CSCs	WPMY-1	MMPs-2, 9, 13, RANKL	Promotes fibroblast growth factor pathway activation, epithelial cell proliferation, differentiation, migration, and the EMT	2015	[82]
miRNA	miRNA-26a	LNCaP, PC-3	LNCaP, PC-3	Not yet researched	Inhibitory effect on EMT process	2019	[83]
miRNA	miR-217,miR-23b-3p	PC-3, DU145	PC-3, DU145	E-Cadherin, N-Cadherin, Vimentin	Promotes the EMT and regulates PC cell proliferation and invasive ability	2020	[84]
miRNA	miR-146a-5p	CAF	LNCaP, DU145	EGFR/ERK	Low expression in CAF promotes EMT and accelerates cancer cell metastasis	2020	[85]
miRNA	miR-95	THP-1, M2-TAMs, PCA	PC3, DU145	JunB	Promotes PC cell proliferation and invasion and the EMT	2020	[86]
circRNA	CIRC_0081234	MDA-PCA-2b	22RV1, DU145	miR-1/MAP3K1 axis	Promotes PC cell migration, invasion, and epithelial transformation	2021	[87]
**Exosomes regulate angiogenesis in PC**							
miRNA	miR-27a-3p	PC-3	HUVEC	Not yet researched	Promotes angiogenesis in endothelial cells	2021	[88]
protein	c-Src, IGF-IR, GRK, FAK	PC-3, DU145, C4-2B	PC-3, DU145, C4-2B	IGF-IR, SrcpY416, GRK5, GRK6	Promotes tumor growth and angiogenesis	2016	[89]
**Exosomes involved in the generation of the tumor microenvironment in PC**							
protein	TGF-β	DU145, PC3	Primary fibroblasts	Smad3	Modulates fibroblast phenotypes and functions	2010	[90]
gene/protein	KRAS, HRAS, RAB	RWPE-1, PC-3, C4-2B	Adipose Stem Cells	Raf	Induces tumorigenic transformation of adipose-derived stem cells	2014	[91]
miRNA	miR-130b	RWPE-1, PC-3, C4-2B	Adipose Stem Cells	Lats2, PDCD4, H-ras, K-ras	Recruits adult stem cells and enhances their clonal expansion through tumor mimicry	2014	[91]
miRNA	miR-125b	RWPE-1, PC-3, C4-2B	Adipose Stem Cells	Lats2, PDCD4, H-ras, K-ras	Recruits adult stem cells and enhances their clonal expansion through tumor mimicry	2014	[91]
miRNA	miR-155	RWPE-1, PC-3, C4-2B	Adipose Stem Cells	Lats2, PDCD4, H-ras, K-ras	Recruits adult stem cells and enhances their clonal expansion through tumor mimicry	2014	[91]
miRNA	miR-375	LNCaP	hFOB1.19	Not specified	Promotes osteoblast activity	2019	[92]
miRNA	miR-409	CAF	normal prostate fibroblasts	2 M, RSU1, STAG2, PHC3, STAG2, NPRL2, RBL2	Promotes the EMT	2014	[93]
miRNA	miR-154, miR-379	ARCaPE, ARCaPM, LNCaP, C4-2	ARCaPE, ARCaPM, LNCaP, C4-2	STAG2, Smad7	Promotes the EMT, cell stemness, and bone metastasis	2014	[94]
Not specified	Not specified	SV-HFO	PC-3	YWHAG, PAK2, CDK5, RAD21 (not verified)	Stimulates tumor cell growth	2015	[95]
LncRNA	lncAY927529	VCaP, LNCaP, DU145, PC3	PC-3, DU145	LC3II, CXCL14	Regulates the bone microenvironment	2021	[96]
**Exosomes contribute to immune escape of PC cells**							
protein	NKG2D ligand	22Rv1	NK, CD8+ T	NKG2D	Downregulation of NKG2D expression on the surface of CD8+ T cells and NK cells	2014	[97]
protein	PGE2	DU145	DC	CD73	Inhibits the presentation of tumor antigens	2017	[98]
miRNA	miR-125a	LNCaP	PBMC	AKT1	Regulates the tumor microenvironment	2014	[99]
protein	CXCR4	RM-1	MDSCs	TLR2/NF- κB	Recruits MDSCs to the tumor microenvironment	2021	[100]
**Exosomes promote PC cell drug resistance**							
protein	Caveolin-1	LNCaP	LNCaP	NFκB	Induces resistance to radiation and chemotherapy	2019	[81]
miRNA	miR-27a	PSC27	PC-3	p53	Mediates chemoresistance in PC-3 cells	2019	[101]
miRNA	miR-423-5p	CAF	LN-CaP, 22Rv-1, C4-2	GREM2	Increases resistance of prostate cancer to taxane	2020	[102]

### Exosomes mediate the EMT in PC

The EMT refers to the transformation of epithelial cells into a quasi-mesenchymal stem cell state in which invasive and metastatic capabilities are acquired while adhesiveness is reduced [103]. A considerable number of recent studies have focused on the role of exosomes in the EMT associated with PC. For example, exosomes containing the integrin α2 subunit enhance focal adhesion kinase (FAK) and ERK1/2 activity in recipient cells to induce the EMT, ultimately promoting the progression of PC into a more aggressive form [79], and exosomes carrying prostate-specific G protein-coupled receptors (PSGRs) have shown similar effects [80]. Furthermore, Lin et al. [81] demonstrated that Cav-1-containing tumor-derived exosomes (TDEs) promoted the EMT in neuroendocrine PC through the NF-κB signaling pathway.

In addition to the aforementioned protein-like content, microRNAs (miRNAs), important cargoes in exosomes, also significantly contribute to the EMT. Both miR-100-5p and miR-21-5p were abundant in exosomes derived from PC cells and promoted the EMT by enhancing the expression of MMP-2, MMP-9, MMP-13, and RANKL [82]. Similarly, miR-26a in exosomes significantly regulated the expression of EMT-related proteins and potently inhibited the proliferation, migration, and invasion of LNCAP and PC-3 cells [83]. Furthermore, the role of miR-217 and miR-23b-3p in upregulating EMT-associated PC aggressiveness has been illustrated [84]. Similar to PC cell-derived exosomes, exosomes derived from other types of cells have shown significant effects on mediating the EMT. For example, miR-146a-5p, the expression of which is decreased in the exosomes derived from cancer-associated fibroblasts, enhanced the EMT by activating the epidermal growth factor receptor (EGFR)/ERK pathway to accelerate cancer cell metastasis [85], while miR-95 was significantly upregulated in exosomes derived from tumor-associated macrophages (TAMs) and promoted the EMT by activating on the downstream gene *JunB* [86]. Constituting another important class of exosomal cargo, circular RNAs (circRNAs) regulated PC progression by sponging miRNAs; e.g., circ_0081234 has been shown to promote PC cell migration and invasion and the EMT via regulating miR-1 [87].

### Exosome regulation of PC angiogenesis

TDEs harbor a variety of angiogenesis-stimulating factors, including vascular endothelial growth factor, fibroblast growth factor, platelet-derived growth factor, basic fibroblast growth factor, transforming growth factor β, tumor necrosis factors α and β, and interleukin 8 [104, 105], which may be involved in the development of PC. MiR-27a-3p in PC-3 cell-secreted exosomes is thought to be potentially involved in the angiogenic behavior of endothelial cells [88]. DeRita et al. [89] reported that exosomes secreted by PC cells are enriched with Src tyrosine kinase, insulin-like growth factor I receptor (IGF-IR), G protein-coupled receptor kinase (GRK), and FAK, all of which play important roles in angiogenesis. Furthermore, TDEs produced under hypoxic conditions have been shown to enhance matrix metalloproteinase activity in premetastatic niches, thereby promoting angiogenesis in PC [106–108].

### Exosomes promote a microenvironment suitable for PC and regulate PC cell immune escape

Tumor cells promote tumor-favoring changes to the microenvironment in affected tissue to induce a series of biological functions in tumor cells, such as maintaining tumor cell characteristics, tumor cell proliferation, and resistance to immune responses and exerting antiapoptotic, and prometastatic effects [109]. The altered tissue microenvironment is called the tumor microenvironment, and it is composed of stromal cells (e.g., pericytes, fibroblasts, and neuroendocrine cells), immune cells (e.g., T and B lymphocytes, dendritic cells (DCs), and macrophages), and the ECM [107]. The interactions between the tumor microenvironment and tumor cells exert a profound effect on tumor development [110], and the intercellular communication function of exosomes and their cargoes in the tumor microenvironment play an important role in maintaining these interactions [107]. Cancer-associated fibroblasts (CAFs) derived from fibroblasts and mesenchymal stem cells have been shown to promote tumor growth [111], and the production and maintenance of CAFs depend on the transforming growth factor-β (TGF-β). In addition, PC cell lines secrete exosomes enriched with surface TGF-β that trigger the SMAD3-related signaling pathway and thus induce CAF phenotype acquisition [90, 112]. Exosomes in metastatic PC not only carry the proto-oncogenes KRAS and HRAS but also transfer their transcription products or RAB proteins to adipose-derived stem cells, transforming them into tumor cells [91]. Three miRNAs (i.e., miR-125, miR-130b, and miR-155) have been shown to induce adipose-derived stem cell tumor reprogramming in tumor-prone patients, and they play important roles in cloning PC cells [91, 113]. Li et al. [92] explored the effects of PC cell-derived exosomes on bone metastasis and found that miR-375 significantly promoted osteoblast activity after exosomes were delivered into osteoblasts. Similarly, lncAY927529 in TDEs is engaged in the establishment of a PC bone metastasis pre-microenvironment [96]. These results suggested that PC cells elicit stromal cell support by shaping stromal cell properties through the action of exosomes, thereby establishing a microenvironment suitable for PC cell survival. In addition to exosomes secreted by tumor cells, exosomes secreted by stromal cells into the microenvironment affect tumors. For example, cancer cells exhibit energy metabolism reprogramming, and therefore, the metabolic state of cancer cells differs from that of normal cells [114], and exosomes secreted by CAFs into the tumor microenvironment are involved in this PC cell metabolic reprogramming [115]. Specifically, these exosomes inhibit mitochondrial oxidative phosphorylation, enhance glycolysis, and even lower the pH of the tumor microenvironment, all outcomes consistent with the Warburg effect and conducive for maintaining the metabolic state of tumor cells and facilitating the survival of PC cells in a hypoxic environment [116]. Josson et al. [93] observed that CAFs transfer miR-409 via exosomes to adjacent epithelial cells, leading to tumorigenesis, the EMT, and epithelial cancer cell stemness. Three miRNAs (miR-209, miR-379, and miR-154) in the DLK1DIO3 region of chromosome 14 have been shown to regulate the EMT and PC bone metastasis [94]. In addition to those secreted by CAFs, exosomes secreted by other stromal cells play important roles in PC. For example, exosomes secreted by mesenchymal-derived osteoblasts exert a proliferative effect on PC-3 cells [95].

Immune cells constitute another important class of cells in the tumor microenvironment, and the immune system composed of them is a main obstacle preventing further development of cancer cells. However, tumors generally develop and progress even in the presence of strict immune system defenses due to tumor cell immune escape [117]. Exosomes mediate communication between tumor cells and immune cells and thus promote immune surveillance evasion. For example, exosomes derived from cancer cells containing FasL [118], TGF-β [119], NKG2D ligand [120], Galectin-9 [121], HSP72 [122] and/or other immunomodulatory factors support the immune escape of tumor cells and induce the differentiation of monocyte cell lines that support the tumor cell phenotype [118]. Studies have provided direct evidence supporting exosome involvement in PC cell immune escape. For example, PC-secreted exosomes expressing NKG2D ligands on the surface induced the downregulated expression of NKG2D on the surface of natural killer (NK) and CD8+ T cells, leading to a decrease in the cytotoxic effect of these killer cells and thus enabling PC cell immune escape [97], whereas a tumor antigen-specific T-cell response produced by DCs was significantly enhanced when Rab27a was added to cells to inhibit exosome release [98]. Furthermore, CD73 expression in DCs was induced by exosomes carrying prostaglandin E2 (PGE2), which inhibited the production of TNFα and IL-12 upon the addition of ATP, ultimately promoting communication between PC cell-derived exosomes, immune cell production and immunosuppression [98]. Kim et al. [99] indicated that the secretion of miR-125a-containing exosomes by PC cells inhibited AKT1 expression in monocytes and macrophages and suppressed their proliferation. Myeloid-derived suppressor cells (MDSCs) exert significant immunosuppressive effects and play key roles in tumor immune escape, and exosomes derived from PC cells promote the recruitment of MDSCs by upregulating chemokine receptor 4 (CXCR4) through the TLR2/NF-κB signaling pathway [100]. In recent years, programmed cell death-ligand 1 (PD-L1) has gained considerable attention. PD-L1 has been found in PC-derived exosomes, and the proliferation and activation of T cells are inhibited, while apoptosis is enhanced, when PD-L1 binds to programmed cell death protein-1 (PD-1) on T cells [123–126]. Furthermore, exosomes can carry PD-L1 to various parts of the body, creating positive conditions for immunosuppression and establishment of a premetastatic microenvironment [127].

### Exosomes are involved in PC drug resistance

The emergence of drug resistance is an important obstacle in the fight against cancer. As important components in intercellular communication, exosomes are crucial to tumor drug resistance [78]. The molecular mechanisms underlying the mediation of tumor drug resistance by exosomes are usually classified into three categories: (1) tumor cells excreting chemotherapeutic drugs through exosomes [105], (2) exosomes carrying resistance cargo that communicates with drug-sensitive tumor cells; and (3) exosomes acting as decoy targets in immunotherapy [78]. In recent years, drug resistance in PC has been attributed to the second category of aforementioned mechanisms, as reported extensively in the literature. For example, PC cells acquire therapeutic resistance to docetaxel through castration-resistant PC (CRPC)-derived exosomes carrying caveolin-1, and many of these cells survive under radiation therapy [81]. Furthermore, exosome-derived miR-27a in PSC27 cells inhibits p53 gene expression and thus mediates the chemoresistance of PC-3 cells [101]. Moreover, CAF-derived exosomes carrying miR-423-5p inhibit GREM2 activity by the regulating the TGF-β pathway and thus increase PC resistance to taxane [102].

## Prospective applications of exosomes in PC diagnostics and treatments

### Exosomes as sources of biomarkers for PC diagnostics

**Fig. 2:**
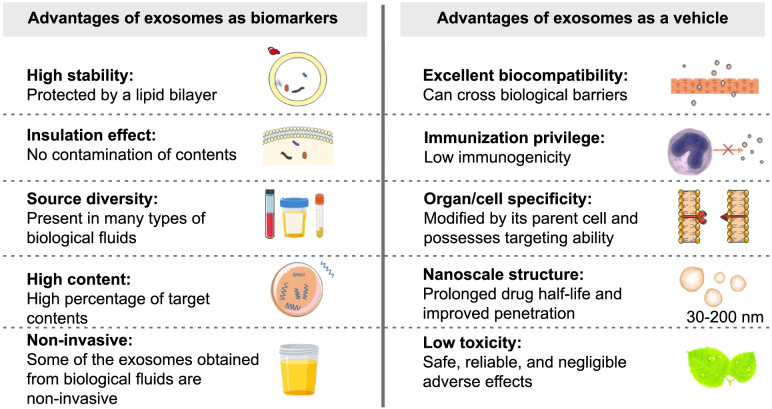
Properties of exosomes as biomarkers and therapeutic vehicles in the diagnosis and treatment of PC

Exosomes are highly stable, and the cell-like topology of their lipid bilayer protects their contents from enzymatic degradation [128]. Notably, exosome cargoes reflect the true primitive state of the parent cells, and the exosomal cargoes never come into contact with bodily fluids, which protects the pathological molecules derived from the secretory cells [129]. Furthermore, exosomes contain large amounts of target contents. For example, the greatest proportion of miRNAs isolated from urine have been shown to be in exosomes, and most of the miRNAs detected in serum and saliva are also found in exosomes [130]. Exosomes are commonly found in most biological fluids, such as plasma [131], urine [132], saliva [133], ascites [134], breast milk [135], and amniotic fluid [136], providing ample sources for investigating their functions, and these sources allow noninvasive or less invasive sampling of bodily fluids compared to solid biopsy sampling, which improves patient compliance and convenience. Due to their optimal properties, exosomes have been established as ideal biomarkers and extensively explored for use in the diagnosis, grading, prognostic assessment, and therapeutic monitoring of PC (Fig. 2; Table 3).

**Table 3:** Application of the contents in exosomes as biomarkers for the diagnosis of prostate cancer (PC)

Source	Isolation method	Identification method	Type	Contents	Application	Ref.
Urine	Precipitation	PCR	lncRNA	lncRNA-p21	Diagnosis of PC	[137]
Urine	Precipitation	PCR	miRNA	miR-21, miR-451, miR-636	Diagnosis of PC	[138]
Urine	UC	Microarray analysis, PCR	miRNA	miR-30b-3p, miR-126-3p	Diagnosis of PC	[139]
Urine	Precipitation	PCR	miRNA	miR-574-3p, miR-141-5p, miR-21-5p	Diagnosis of PC	[140]
Urine	Precipitation	PCR	miRNA	miRNA-375, miRNA-574-3p	Diagnosis of PC	[141]
Urine	UC	NGS, PCR	miRNA	miR-196a-5p, miR-501-3p	Diagnosis of PC	[142]
Urine	Precipitation	PCR	miRNA	miR-2909	Diagnosis of PC	[143]
Urine	UC	Proteomics, WB	protein	ITGA3, ITGB1	Diagnosis of PC	[144]
Urine	UC	Proteomics, WB, ELISA	protein	Flotillin 2, TMEM256, Rab3B, LAMTOR1, Park7	Diagnosis of PC	[145]
Urine	UC	Lipidomics	lipidome	Phosphatidylserine, lactosylceramide	Diagnosis of PC	[146]
Plasma	Precipitation	PCR	lncRNA	SAP30L-AS1, SChLAP1	Diagnosis of PC	[147]
plasma	Precipitation	PCR	miRNA	mIR-125a-5p, miR-141-5p	Diagnosis of PC	[148]
Serum	Precipitation	PCR	miRNA	miR375, miR21, miR574	Diagnosis of PC	[149]
Serum	UC	WB, ELISA	protein	ephrinA2	Diagnosis of PC	[150]
Plasma, Serum	UC	Infrared spectroscopy, Raman spectroscopy	protein	Alpha-helical proteins, beta-folded proteins	Diagnosis of PC	[151]
Serum	UC, Magnetic beads	Proteomics, WB	protein	Gamma-glutamyltransferase	Diagnosis of PC	[152]
Serum		Microfluidic Raman biochip	protein	EpCAM of exosomes	Diagnosis of PC	[153]
Plasma	UC	Proteomics, WB, ELISA	protein	Claudin 3	Diagnosis of PC	[154]
Urine	Precipitation	PCR	lncRNA	PCGEM1, PCA3	Diagnosis of high-grade PC	[155]
Serum	Precipitation	PCR	miRNA	miR-141, miR-375	Diagnosis of high-grade PC	[156]
Serum	Precipitation	PCR	miRNA	miR-1246	Diagnosis of high-grade PC	[157]
Plasma	Precipitation	RNA-Seq, PCR	miRNA	miR423-3p	Diagnosis of high-grade PC	[158]
Urine	UC	Proteomics, WB	protein	FABP5	Diagnosis of high-grade PC	[159]
Urine	Precipitation	Microarray analysis, PCR	sncRNA	A set of sncRNAs	Staging PC	[160]
Plasma	UC	WB, ELISA	protein	Survivin	Diagnosis of early PC	[161]
Plasma	Density gradient centrifugation, antibody beads	WB	protein	αvβ3 Integrin	Monitoring PC progression	[162]
Plasma	Precipitation	RNA-Seq, PCR	miRNA	miR-1290, miR-375	Prognosis in CRPC	[163]
Plasma	UC, Precipitation, antibody-bead	PCR	mRNA	AR-V7	Prognosis in CRPC	[164]
Serum	Precipitation	RNA-Seq	miRNA	miR-654-3p, miR-379-5p	Treatment effect observations	[165]
Serum	UC	PCR	mRNA	CD44v8-10 mRNA	Drug resistance monitoring	[166]
Urine	UC	Proteomics, WB	protein	TM256/LAMTOR1	Diagnosis of PC	[167]
Urine	UC	Microarray analysis, WB	protein	ADSV/TGM4	Staging PC	[168]
Urine	UC	Microarray analysis, WB	protein	CD63/GLPK5/SPHM/PSA/PAPP	Staging PC	[168]
Semen	UC	PCR	miRNA	miR-142-3p/miR-142-5p/miR-223-3p	Diagnosis/prognosis in PC	[169]

Both urine and blood samples have been extensively investigated because they are conveniently collected and readily available. Studies have revealed significant quantitative differences between urinary exosome contents obtained from PC patients and from benign prostatic hyperplasia (BPH) patients (or healthy individuals); for example, differences have been found in the quantities of lncRNA-p21 [137], miR-21, miR-451, miR-636 [138], miR-30b-3p, miR-126-3p [139], miR-574-3p, miR-141-5p, miR-21-5p [140], miRNA-375 [141], miR-196a-5p, miR-501-3p [142], miR-2909 [143]; proteins such as ITGA3, ITGB1 [144], flotillin 2, TMEM256, Rab3B, LAMTOR1, and Park7 [145]; and some lipid species such as phosphatidylserine and lactosylceramide [146]. Furthermore, the diagnostic potential of exosomes obtained from PC patient blood has been explored. For example, the levels of nucleic acid molecules, such as SAP30L-AS1, SChLAP1 [147], miR-125a-5p, miR-141-5p [148], miR375, miR21, and miR574 [149], and proteins, such as ephrinA2 [150], alpha-helical proteins and beta-folded proteins [151], gamma-glutamyltransferase [152], EpCAM [153], Claudin 3 [154], and other proteins, are significantly increased in PC patients, and variations in these levels in exosomes are expected to show diagnostic potential in PC. Furthermore, exosomal contents play important roles in PC staging, early diagnosis, progression tracking, prognostic assessment, and treatment monitoring. For example, a group of molecules, including PCGEM1, PC3 [155], miR-141 [156], miR-1246 [157], miR-375, miR423-3p [158], and FABP5 [159], and a series of small noncoding RNAs (sncRNAs) [160] have shown diagnostic potential in high-grade PC and even usefulness in PC staging. The nondifferential increase in the Gleason score of survivin in the plasma exosomes of PC patients suggested its potential for use in early PC diagnosis [161]. Furthermore, exosomal αvβ3 integrin in PC patients is a promising biomarker for use in tracking PC progression [162], and a group of predictors of prognostic status in patients with CRPC have been identified, including miR-1290, miR-375 [163] and AR-V7 [164]. Moreover, two miRNAs (i.e., miR-654-3p and miR-379-5p) in exosomes can be potentially used to predict the efficacy of carbon ion radiation therapy (CIRT) for the treatment of PC [165], and CD44v8-10 mRNA in exosomes can be used as a diagnostic marker for docetaxel-resistant CRPC [166].

In comparison to a single variable used as a diagnostic marker, a combination of exosomal cargoes can significantly improve diagnostic performance and create novel diagnostic opportunities. For example, the PCA3/PCGEM1 combination has significantly improved the accuracy of high-grade PC diagnoses, and the TM256/LAMTOR1 combination is highly sensitive for diagnosing PC in patients [167]. The combination of ADSV and TGM4 can be used to distinguish benign tumors from malignant prostate tumors. The ExoDx Prostate IntelliScore from Exosome Diagnostics, which is based on the detection of ERG, PCA3, and SPDEF RNA in urinary exosomes, can be used to distinguish high-grade PC from low-grade PC and benign disease, and the combination of five proteins (i.e., CD63, GLPK5, SPHM, PSA, and PAPP) able to serve a similar purpose [168, 170, 171]. Furthermore, the semen exosome-based PSA/miR-142-3p/miR-142-5p/miR-223-3p model has shown potential for improving PC diagnostic/prognostic efficiency [169].

### Exosomes as potential therapeutic targets in PC treatment

To date, rapidly advancing drug delivery technologies have led to the development of many nanoscale drug delivery systems (DDSs), which show significant promise for improving targeted therapies. Unfortunately, induced toxicity and/or the mononuclear phagocyte system has prevented the clinical transformation of many DDSs [172]. However, exosomes may provide the ultimate solution to these problems (Fig. 2). In addition to their high permeability and capacity for prolonging drug half-lives due to their nanoscale structure [173], exosomes are hypotoxic compared to other DDSs [174]. The highly homologous nature of exosomes, particularly their parent cell-derived lipid membranes, endows them with immune privilege, improving the odds of exosomes evading phagocytosis by immune cells [175]. Moreover, the targeting ability conferred by modifications and innate biological barrier permeability significantly facilitate exosome translocation to desired target areas [172, 176–178]. Because of these optimal properties, exosomes have been extensively explored as potential therapeutic vehicles for PC treatment. For example, Saari et al. [179] introduced exosomes carrying paclitaxel into both LNCaP and PC-3 cell cultures and found that these exosomes exerted a cytotoxic effect after endocytosis by target cells. Although it has been shown that natural exosomes can be used as therapeutic carriers, various problems, such as insufficient targeting ability [180], have limited the clinical applications of these exosomes as therapeutic carriers. Therefore, it is important to improve the targeting properties of natural exosomes by enhancing their targeting ability. Pan et al. [181] developed a targeted therapeutic platform using urinary exosomes obtained from PC patients to develop PMA/Iron-HSA@DOX nanoparticles that target EGFR on tumor cells and downstream AKT/NF-kB/IkB signaling; notably, the high safety profile of this homologous infusion technique, which did not induce cellular inflammation, shows significant promise for clinical application. Similar to this is the modification of mesenchymal stem cell-associated exosomes (MSCs-Exo) by altanerova et al. Superparamagnetic iron oxide nanoparticles inside the piggybacked MSCs-Exo produced toxicity on PC3 cells after external alternating magnetic field-induced thermotherapy [182]. Furthermore, Vázquez-Ríos et al. [183] designed a nanoplatform loaded with oncology therapeutics that mimicked exosomes, and these nanoplatforms showed efficient targeting capability, similar to that of tumor-associated exosomes. Moreover, Severic et al. [184] added PSMA-targeting peptides to the surface of exosome mimics to target PSMA-positive PC cell lines (i.e., the LNCaP and C4-2B cell lines), and these mimics showed superior targeting ability both in vivo and in vitro.

Due to the significant roles that exosomes play in promoting PC progression and mediating immunosuppression, exosome-based vaccines have been developed to block tumor progression signaling. For example, exosomes secreted by DCs have been used to develop an effective cell-free peptide-based vaccine that leverages MHC class I/peptide complex contributed by DCs to induce CD8+ T cells to eradicate tumor cells [185]. Furthermore, Shi et al. [186] developed an exosome vaccine by anchoring interferon-gamma fusion proteins to the surface of exosomes derived from PC cells, and subsequent validation experiments revealed that the number of M1 macrophages was increased and exosome phagocytosis was enhanced, while the concentration of antibodies against the exosomes was increased and the expression of vascular endothelial growth factor receptor 2 was downregulated.

Although the superior performance of exosomes compared to other nanocarriers has been shown, the disadvantageous properties of exosomes, e.g., low volume, high heterogeneity, complex cargo, and difficulty in characterization, has ultimately made it challenging to apply exosomes in clinical practice [178].

## Conclusions

In recent years, as our understanding of exosomes and PC continued to rapidly grow, an increasing number of molecular mechanisms underlying exosomal involvement in PC progression was discovered. Both tumor cell-derived and stromal cell-derived exosomes were revealed to be important factors in PC progression. A significant amount of effort has been devoted to investigating the molecular mechanisms regulating the exosomal protein-like and nucleic acid-like content in inducing the EMT, tumor angiogenesis, tumor microenvironment establishment, and drug resistance, leading to tumor progression. The clarification of these mechanisms provides the precedence for further exploration into diagnostic markers and the development of effective drugs for PC prevention and treatment. As common nanocarriers, exosomes show the following inherent advantages compared to other nanocarriers: Exosomes are highly stable in biological fluids, exhibit excellent protection of their cargo, can encapsulate high concentrations of biomarkers, and can be used to differentiate tumor risk, subtype, recurrence likelihood, and progression based on their molecular contents, and they can be obtained through noninvasive procedures (i.e., in urine specimens). Undoubtedly biomarkers with these optimal properties offer significant promise for effective screening, diagnosis, prognosis, and treatment monitoring of PC in clinical applications. Fortunately, some of these markers have been tested in clinical trials. In addition to potential diagnostic markers of PC, exosomes have shown various advantages, including biological barrier permeability, prolonged circulation time, low toxicity, low immunogenicity, and modifiability, that endow them with significant advantages for use as therapeutics carriers. Substantial advancements have been made in using exosomes, including natural and engineered exosomes, as models to develop and improve targeted DDSs.

Although exosomes have a bright future in medical applications, many unsolved problems remain; e.g., the molecular mechanisms regulating the involvement of exosomes in PC remain unclear. Furthermore, current techniques for exosome isolation and identification require large samples and are expensive, ultimately hindering their clinical applications. Moreover, recent experiments have been mostly conducted at the cellular and molecular levels, and large-scale and multicentered in vivo experiments are needed to provide evidence for the safe and efficient applications of exosomes in clinical settings.

## Data Availability

Not applicable.

## Abbreviations

PC: Prostate cancer
PSA: Prostate-specific antigen
PHI: Prostate health index
PCA3: Prostate cancer antigen 3
MVB: Multivesicular body
ESE: Early sorting endosome
ER: Endoplasmic reticulum
TGN: Trans-Golgi network
LSE: Late sorting endosome
ILV: Intraluminal vesicle
ESCRT: Endosomal sorting complex required for transport
ALIX: Apoptosis-linked gene 2-interacting protein x
Arf6: ADP-ribosylation factor 6
PLD2: Phospholipase D2
NSMase2: Neutral sphingomyelinase 2
TSG101: Tumor susceptibility 101
PIKfyve: Phosphoinositide kinase
YKT6: YKT6 v-SNARE homolog
SNARE: Small NF90 (ILF3) associated RNA E
EMT: Epithelial-mesenchymal transition
PSGR: Prostate-specific G-protein coupled receptor
TDE: Tumor-derived exosome
EGFR: Epidermal growth factor receptor
TAM: Tumor-associated macrophage
VEGF: Vascular endothelial growth factor
IGF-IR: Insulin-like growth factor–I receptor
GRK: G protein-coupled receptor kinase
FAK: Focal adhesion kinase
ECM: Extracellular matrix
CAF: Cancer-associated fibroblast
TGF-β: Transforming growth factor-β
SMAD3: SMAD family member 3
Fasl: Fas ligand
DCs: Dendritic cells
PGE2: Prostaglandin E2
TNFα: Tumor necrosis factor α
ATP: Adenosine triphosphate
PBMC: Peripheral blood mononuclear cells
AKT1: AKT serine/threonine kinase 1
MDSCs: Myeloid-derived suppressor cells
TLR2: Toll-like receptor 2
CXCR4: C-X-C motif chemokine receptor 4
PD-L1: Programmed cell death 1 ligand 1
PD-1: Programmed cell death-1
GREM2: Gremlin 2
miRNA: MicroRNA
lncRNA: Long non-coding RNA
ITGA3: Integrin subunit alpha 3
ITGB1: Integrin subunit beta 1
TMEM256: Transmembrane protein 256
LAMTOR1: MAPK and MTOR activator 1
Park7: Parkinsonism associated deglycase
SAP30L-AS1: SAP30L antisense RNA 1
SCHLAP1: SWI/SNF complex antagonist associated with prostate cancer 1
EpCAM: Epithelial cell adhesion molecule
PSMA: Proteasome 20S subunit alpha
PCGEM1: PCGEM1 prostate-specific transcript
FABP5: Fatty acid binding protein 5
sncRNA: Small noncoding RNA
CIRT: Carbon ion radiation therapy
CRPC: Castration-resistant prostate cancer
TM256: Transmembrane protein 256
TGM4: Transglutaminase 4
DDS: Drug delivery system
MHC: Major histocompatibility complex
